# Emu-miR-10a-5p in *Echinococcus multilocularis*-derived-extracellular vesicles alleviates airway inflammation in mice with allergic asthma by inhibiting macrophage M2a polarization through LIF-mediated JAK1–STAT3 signaling

**DOI:** 10.3389/fimmu.2025.1577349

**Published:** 2025-05-27

**Authors:** Yunzhuo Xin, Rou Wen, Dong Song, Jing Xiao, Xiaoping Gao, Mei Yin, Yanli Bai, Jie Wang, Xiangyu Zhou, Jiaqing Zhao

**Affiliations:** ^1^ School of Basic Medicine, Ningxia Medical University, Yinchuan, China; ^2^ Department of Blood Transfusion, Xi’an International Medical Center Hospital, Xi’an, China; ^3^ Scientific Technology Center of Ningxia Medical University, Yinchuan, China; ^4^ Ningxia Key Laboratory of Prevention and Control of Common Infectious Diseases, Ningxia Medical University, Yinchuan, China; ^5^ Department of Otolaryngology Head and Neck Surgery, General Hospital of Ningxia Medical University, Yinchuan, China; ^6^ Clinical Laboratory, Shiyan Integrated Traditional Chinese and Western Medicine Hospital, Shiyan, China; ^7^ Department of Respiratory Medicine, General Hospital of Ningxia Medical University, Yinchuan, China; ^8^ Center for Neurological Diseases, The First Hospital of Shizuishan, Affiliated of Ningxia Medical University, Shizuishan, China

**Keywords:** asthma, extracellular vesicle, leukemia inhibitory factor (LIF), M2a macrophage, emu-miRNA-10a-5p

## Abstract

**Introduction:**

Parasites and parasite-derived extracellular vesicles (EVs) and microRNAs (miRNAs) can protect against inflammatory diseases, such as asthma. M2a macrophages facilitate the development of allergic asthma. miR-10a-5p is closely associated with asthma, and emu-miR-10a-5p (encapsulated in *Echinococcus multilocularis* EVs), shares seed-site sequences with mature human and mouse miRNAs.

**Methods:**

We purified EVs by centrifugation, and characterized the EVs via nanoparticle tracking analysis (NTA) and transmission electron microscopy (TEM). We used MH-S cells to construct the M2a polarization model. The gene expression changes in MH-S cells after transfect with emu-miR-10a-5p mimics was analyzed through transcriptome sequencing. We established a mouse model of ovalbumin (OVA)-induced allergic asthma. Hematoxylin and eosin (H&E), Masson’s trichrome, and periodic acid–Schiff (PAS) staining were used to detect airway inflammation in the lung tissues. RT-qPCR, flow cytometry and Western Blot assays were performed to validate the expression of related genes and proteins.

**Results:**

Here, we observed that *E. multilocularis*-derived EVs and their encapsulated emu-miR-10a-5p transcripts inhibited macrophage M2a polarization. We also found that emu-miR-10a-5p targeted leukemia inhibitory factor (LIF) mRNA and inhibited the downstream Janus kinase 1 (JAK1)–signal transducer and activator of transcription 3 (STAT3)-signaling pathway. We established a mouse model of ovalbumin (OVA)-induced allergic asthma and found that emu-miR-10a-5p alleviated pulmonary inflammation in mice with allergic asthma while inhibiting the accumulation of pulmonary M2a macrophages. emu-miR-10a-5p intervention inhibited LIF and JAK1–STAT3 signaling in the lungs of mice with allergic asthma.

**Discussion:**

These findings suggest that emu-miR-10a-5p encapsulated in *E. multilocularis* EVs might regulate M2a macrophage polarization via the JAK1–STAT3 pathway by targeting and binding LIF in a cross-species manner, thereby alleviating airway inflammation in mice with allergic asthma. These findings enhance the current understanding of the mechanisms underlying immune regulation during infection and the maintenance of immune stability.

## Introduction

Asthma is the most common chronic respiratory disease, affecting approximately 334 million people worldwide, with a high incidence in developed countries (1). Allergic asthma is the most common form of asthma (2). Although glucocorticoids can reduce asthma-related morbidity and mortality, the global impact of asthma remains high, and the prevalence of the disease is apparently increasing in developing countries (3, 4).

Macrophages are the most abundant immune cells in the lungs and play a key role in maintaining the immune response to respiratory inflammation (5, 6). Macrophages can be polarized into classically activated (M1) and alternatively activated (M2) phenotypes, corresponding to Th1 and Th2 differentiation of helper T cells, respectively (7). Increased M2 macrophage activation is thought to play a key role in allergic asthma by activating the Th2 cell response (7–9). M2 macrophages are further subdivided into M2a, M2b, M2c, and M2d subtypes, with M2a macrophages being the most closely associated with asthma (10, 11).

Parasites can regulate the host immune system to evade immune rejection, a strategy that promotes the long-term survival of parasites and leads to chronic infections (12). Such regulation can also protect the body from inflammatory diseases caused by dysregulated immune responses (13). Research has shown that some worm infections confer protection against allergic diseases, such as asthma (14). For example, *Schistosoma* infection inhibits the development of airway inflammation in mice with allergic asthma, and *Echinococcus granulosus* infection significantly reduces ovalbumin (OVA)-induced eosinophilic infiltration and mucus production in the bronchoalveolar lavage fluid of mice with allergic asthma and improves airway hyperresponsiveness (15–17). Extracellular vesicles (EVs) are key factors in host–parasite interactions. EVs, approximately 30–150 nm in diameter (18), are secreted by all living cells. EVs typically transport proteins, lipids, and nucleic acids and can potentially serve as immunomodulatory targeting host cells (19). EVs can become internalized by multiple host cells and can regulate host immune responses through the bioactive substances they carry, such as proteins, lipids, and nucleic acids. EVs are rich in non-coding RNAs, especially microRNAs (miRNAs), which specifically bind to target mRNAs, leading to mRNA degradation or inhibiting protein translation, ultimately downregulating targeted proteins. Parasitic miRNAs exist in animal and human bodily fluids and regulate host genes in a cross-species manner (20). *E. granulosus*-derived EVs and encapsulated egr-miR-277a-3p promoted dendritic cell (DC) maturation and differentiation in a cross-species manner, thereby regulating the host immune response (21). Emu-miR-4989-3p encapsulated in the EVs of *E. multilocularis* played a role in nitric oxide production in macrophages, tumor necrosis-alpha production, and the production of several key components involved in the lipopolysaccharide–Toll-like receptor 4 signaling pathway, thereby participating in immunomodulation (22). However, the roles of miRNAs derived from *E. multilocularis* EVs in allergic asthma have not been elucidated.

Sera from mice infected with *E. multilocularis* contained emu-miR-10a-5p (23), suggesting that emu-miR-10a-5p can be delivered to the host. Emu-miR-10a-5p shares seed-site sequences with mature miRNAs from both human and mouse sources and may alter the expression of the corresponding host mRNA, signaling a potential advantage for parasite invasion. Therefore, we speculate that emu-miR-10a-5p may participate in parasite infection and immune responses and play important roles in host–parasite interactions. In addition, previous data suggest that miR-10a-5p is closely associated with asthma and may be a candidate for treating asthma (24, 25).

In this study, we found that EVs and emu-miR-10a-5p secreted by *E. multilocularis* act on the JAK1–STAT3 signaling pathway by targeting and binding to leukemia inhibitory factor (LIF), inhibiting M2a macrophage polarization and thereby alleviating OVA-induced allergic asthma.

## Materials and methods

### Ethics statement

Our research on experimental animals was authorized by the Ethics Committee of Ningxia Medical University (license number: SYXK2020–0001). The data obtained met the relevant ethical requirements.

### Animals and treatments

BALB/c female mice, aged 6–8 weeks and weighing 18–25 g, were purchased from Beijing HFK Bioscience (license number: SCXK (Beijing) 2024–0003). All mice were housed in a specific pathogen-free environment. Mouse experiments and euthanasia procedures were conducted in accordance with the animal welfare guidelines of Ningxia Medical University.

**Figure 1:**
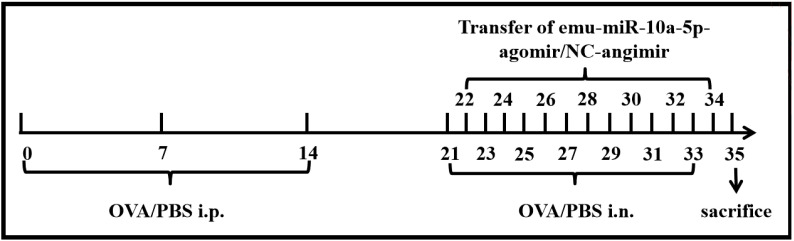
Construction of animal models.

The mice were randomly divided into four groups: the control, OVA, OVA +NC-agomir, and OVA+emu-miR-10a-5p-agomir groups. Asthma was induced and treated as shown in **Figure 1**. Briefly, all mice were sensitized with 200 μL sensitizing solution: 20 μg OVA (Sigma-Aldrich, USA) and 50 μL Al(OH)_3_ (Thermo Fisher Scientific, Xian, China) via intraperitoneal injection on days 0, 7, and 14. On days 21, 23, 25, 27, 29, 31, and 33, nasal drops were administered with 20 μL OVA (100 μg) solution. On days 22, 24, 26, 28, 30, 32, and 34, agomirs were injected via the tail vein at a concentration of 3 OD (dissolve in 200 μL DEPC water). The emu-miR-10a-5p and control agomirs were designed and synthesized by GenePharma (Shanghai, China).

### Isolation and characterization of EVs

PSCs (n = 2,000) were cultured in Dulbecco’s modified Eagles’ medium (DMEM; Gibco, Thermo Fisher Scientific) containing 30% exosome-depleted fetal bovine serum (VivaCell, Shanghai, China) and 1% penicillin–streptomycin in 100 cm^2^ cell culture dishes at 37°C and 5% CO_2_. The PSC culture medium was harvested every 72 h. The medium was collected and centrifuged sequentially at 1500, 2000, 10,000, and 120,000 × *g* for 30, 30, 60, and 90 min, respectively. All centrifugations were carried out at 4°C. The purified EVs were resuspended in phosphate-buffered saline (PBS) and stored at -80°C for subsequent experiments. We measured EV particle sizes and concentrations via nanoparticle tracking analysis (NTA) and characterized the EVs via transmission electron microscopy (TEM) as described previously (26).

### Cell culture

MH-S cells (mouse alveolar macrophages) were procured from ProCell (CL-0597, Wuhan, China). Cells were cultured in RPMI-1640 medium (Gibco, Thermo Fisher Scientific) containing 10% serum (Gibco, Thermo Fisher Scientific), 0.05 mM β-mercaptoethanol, and 1% penicillin–streptomycin (Solarbio Science & Technology, Beijing, China) at 37°C with 5% CO_2_. M2a polarization in MH-S cells was induced by interleukin (IL)-4 (20 ng/mL; BioLegend, USA) and IL-13 (20 ng/mL; BioLegend, USA). The emu-miR-10a-5p mimic, LIF small-interfering RNA (siRNA), and negative controls (NCs) were designed and synthesized by GenePharma (Shanghai, China). Cells were transfected using Lipofectamine 3000 (Invitrogen, USA) according to the manufacturer’s instructions.

HEK293T cells were cultured in DMEM (Gibco, Thermo Fisher Scientific) containing 10% serum and 1% penicillin–streptomycin at 37°C with 5% CO_2_.

### Isolating naïve cluster of differentiation 4+ T cells

Mice were anesthetized, sacrificed, and immersed in 75% alcohol, after which their spleens were aseptically obtained. Lymphocytes were isolated using a Mouse Spleen Lymphocyte Isolation Medium Kit (Tianjin Haoyang Biopharmaceutical, China). Naïve CD4^+^ T cells were purified using a Mouse Naïve CD4^+^ T Cell Isolation Kit (Miltenyi Biotec, Germany).

### Culturing naïve CD4+ T cells with MH-S cells

Naïve CD4^+^ T cells were cultured in RPMI-1640 medium (Gibco, Thermo Fisher Scientific) containing 10% serum (Gibco, Thermo Fisher Scientific) and 1% penicillin–streptomycin (Solarbio Science & Technology) at 37°C with 5% CO_2_. Each 24-well plate (coated with anti-CD3, 1 mg/mL; Thermo Fisher Scientific) contained 5 × 10^5^ naïve CD4^+^ T cells, 1 × 10^5^ MH-S cells treated under different conditions, and soluble anti-CD28 (0.2 mg/mL, Thermo Fisher Scientific). The phenotype of the naïve CD4^+^ T cells was observed via flow cytometry after 3 days.

### Tissue staining and flow cytometry

Lung and spleen tissues were processed into single-cell suspensions. Next, the concentration was adjusted to 1 × 10^7^ cells/mL, and 100 μL of cells was taken from each tube for staining. Lung macrophages were incubated with antibodies against EGF-like module-containing mucin-like hormone receptor-like 1 (F4/80), CD11c, and CD86 at 4°C for 30 min; fixed at 25°C for 30 min and finally incubated with a membrane-breaking solution and an anti-CD206 antibody at 4°C for 30 min. MH-S cells were collected after 48 h of culture, incubated with anti-F4/80 at 4°C for 30 min, fixed at 25°C for 30 min, and finally incubated with membrane-breaking solution and anti-CD206 at 4°C for 30 min. To stain lung eosinophils, we incubated them with antibodies against CD45, CD11C, and sialic acid binding Ig-like lectin F (siglec-F) for 30 min at 4°C. Th1/2 cells were stained by first preparing them as single-cell suspensions and incubating them with cell activation cocktail (BioLegend, USA) for 5 h at 37°C in a 5% CO_2_ incubator. Then, the cells were incubated on ice for 30 min with antibodies against CD3 and CD4, fixed at 25°C for 30 min, and finally incubated on ice for 30 min with membrane-breaking solution, anti-interferon-γ and anti-IL-4. Staining was followed by detection with a FACSCelesta instrument (Becton Dickinson, Beijing, China) and analysis with FlowJo software.

The following reagents for flow cytometry were obtained from BioLegend (USA): PE-anti-F4/80, APC-anti-CD206, FITC-anti-CD11c, APC-anti-siglec-F, APC-anti-CD3, FITC-anti-CD4, PE-anti-IL-4, PerCp-cy5.5-anti-IFN-γ, Cell Activation Cocktail with Brefeldin A, and Intracellular Staining Permeabilization Wash Buffer. We also obtained BV421-anti-CD86 and APC-cy7-anti-CD45 from BD Pharmingen (USA).

### Enzyme-linked immunosorbent assay analysis

After the mice were anesthetized and sacrificed, their eyeballs were removed to obtain blood, and the serum was separated via centrifugation. OVA-specific IgE was detected using a mouse OVA-specific IgE (OVA-sIgE) ELISA Kit (Nanjing Boyan Biotechnology, China) according to the manufacturer’s protocol.

### Reverse transcription-quantitative polymerase chain reaction analysis

Total RNA was extracted from cells using TRIzol reagent (Thermo Fisher Scientific). Total RNA was extracted from mouse lung tissue using an RNAprep Pure Tissue Kit (TianGen, Beijing, China). Reverse transcription of mRNA was performed using PrimeScript™ RT Master Mix (Takara Biomedical Technology, Beijing, China). mRNA-expression levels were detected using Bestar^®^ SYBR Green qPCR Master Mix (DBI^®^ Bioscience). miRNAs were reverse transcribed and detected using the All-in-One miRNA RT-qPCR Detection Kit (GeneCopoeia, Rockville, MD). U6 small nuclear RNA and glyceraldehyde-3-phosphate dehydrogenase RNA were detected as endogenous references and gene expression was calculated using the 2^-ΔΔCT^ method. The primer sequences are shown in **Supplementary Table S1**.

### Western blot analysis

The total protein in MH-S cells was extracted using a Total Protein Extraction Kit (KeyGEN, Nanjing, China), and the concentrations were measured using a BCA Protein Assay Kit (KeyGEN). Antibodies against LIF (ab113262, 1:1000), JAK1 (ab133666, 1:2000), and STAT3 (ab68153, 1:2000) were obtained from Abcam (Shanghai, China), Antibodies against CD9 (13174, 1:1000), CD63 (52090, 1:1000), p-JAK1 (74129, 1:1000), and p-STAT3 (9145, 1:1000) were obtained from (Cell Signaling Technology, Inc., Danvers, Massachusetts, USA). An antibody against β-actin (BS6007MH, 1:10000) and the secondary antibody (BS20241-Y, 1:20000) was obtained from Bioworld Technology (Minneapolis, MN, USA). Protein-expression assays were performed using the ECL Detection Kit (KeyGen Biotech) and the ChemiDoc Touch Imaging System (Bio-Rad Laboratories, Shanghai, China).

### Dual-luciferase reporter assay

Direct association of LIF transcripts with emu-miR-10a-5p was verified using the Luciferase-3′-UTR Reporter System. The complete fragment of the LIF 3′-untranslated region (UTR) located downstream of the Renilla luciferase coding sequence (XhoI/NotI site) was cloned into the psiCHECK-2 plasmid (Promega, Shanghai, China). The resulting plasmid was then co-transfected with the emu-miR-10a-5p mimic or NC (50 ng) into HEK293T cells. The cells were incubated at 37°C for 48 h and collected. Firefly and Renilla luciferase activities were measured using the Dual Glo Dual Luciferase Assay System (Promega, Shanghai, China). For each sample, the firefly luciferase activity was normalized to that of Renilla luciferase.

### Histopathologic analysis

Left lung tissues were fixed in 10% formalin and embedded in paraffin. Hematoxylin and eosin (H&E), Masson’s trichrome, and periodic acid–Schiff (PAS) staining were used to detect airway inflammation, collagen deposition, and mucus production in the lung tissues.

### Immunofluorescence analysis

For IF analysis, paraffin-embedded blocks were processed as previously described (27). Primary antibodies against F4/80 (ab300421, 1:500, Abcam), arginase 1 (Arg1; TD6657, 1:500, Abmart, Shanghai, China), and LIF (ab113262, 1:500, Abcam) were added to the blocks dropwise, and then they were incubated at 4°C overnight. On the following day, the blocks were rinsed five times with PBS, Cy3-conjugated goat anti-rabbit (1:200; Proteintech, China) was added dropwise, and the blocks were incubated at 37°C for 1.5 h, avoiding light. Finally, the nuclei were stained with 4', 6-diamidino-2-phenylindole (Beyotime, China) and imaged using a panoramic slice scanner (PANNORAMIC MIDI, 3DHISTECH, Hungary).

### Statistical analysis

All data were analyzed using GraphPad Prism Software 8.0 (GraphPad Software) and processed as mean ± the standard deviation. Student’s *t*-test was used to analyze differences in the data from the two groups, and differences were considered statistically significant at *P* < 0.05.

## Results

### Identification and characterization of EVs

**Figure 2:**
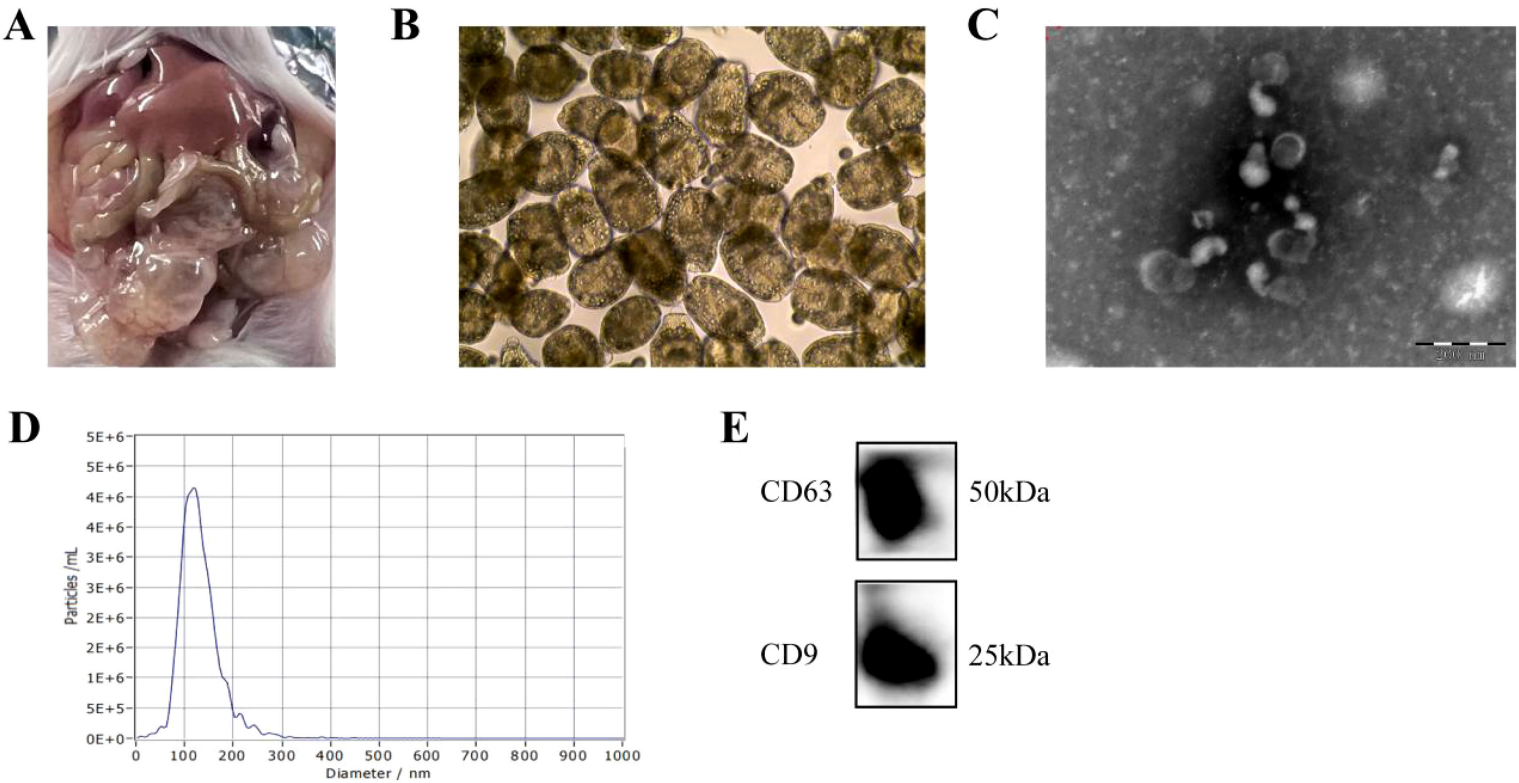
Identification and characterization of EVs secreted by *E. multilocularis*. **(A)** Anatomical view of protoscolex intraperitoneally infected mice. **(B)** Observation of the PSC under the light microscope. **(C)** TEM images of EVs. Scale bars = 200 nm. **(D)** The size distribution of EVs was analyzed via NTA. **(E)** The encapsulation of CD9 and CD63 in EVs was determined using western blotting.

*E. multilocularis* Qinghai isolate (Chinese mainland strain, Qinghai population) was maintained in BALB/c mice in our laboratory. Protoscoleces (PSCs) were obtained from these mice (**Figures 2A, B**). To confirm that EVs were successfully isolated, their sizes and morphologies were characterized via TEM and NTA. Vesicles with round or oval membranes less than 200 nm in diameter were observed via TEM (**Figure 2C**), and the main peaks of the vesicles were between 100 and 130 nm (**Figure 2D**). Our western blot results confirmed the presence of the EV-marker proteins, CD63 and CD9 (**Figure 2E**).

### *E. multilocularis* EVs and encapsulated emu-miR-10a-5p regulated M2a macrophage polarization

**Figure 3:**
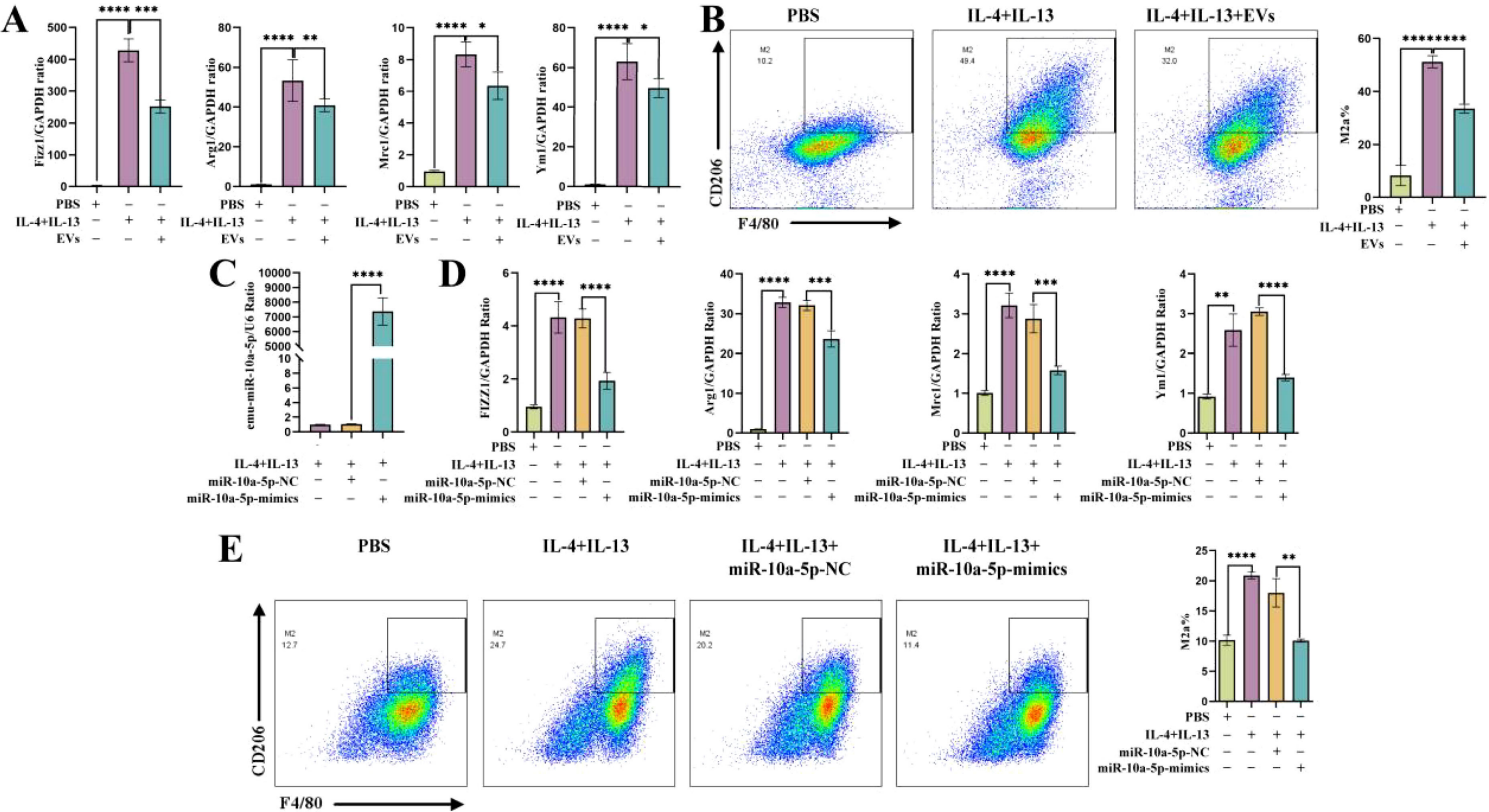
*E. multilocularis* EVs and encapsulated emu-miR-10a-5p regulated M2a macrophage polarization. **(A)** mRNA-expression levels of *Fizz1*, *Arg1*, *Mrc1*, and *Ym1* in macrophages after co-culturing them with EVs, as determined using RT-qPCR. **(B)** Flow cytometry was used to detect M2a-type macrophages after co-culture with EVs. **(C)** emu-miR-10a-5p expression detected using RT-qPCR. **(D)** mRNA-expression levels of *Fizz1*, *Arg1*, *Mrc1*, and *Ym1* in macrophages transfected with emu-miR-10a-5p mimics, as determined via RT-qPCR. **(E)** Flow cytometry was performed to detect M2a-type macrophages after transfection with emu-miR-10a-5p mimics. Except **(B)** n=3, the remaining experiments n=9. *P < 0.05, **P < 0.01, ***P < 0.001, ****P < 0.0001.

We used IL-4 and IL-13 to induce M2a polarization in MH-S cells and co-cultured them with EVs. Our RT-qPCR results revealed lower expression levels of M2a genes (found in inflammatory zone 1 [*Fizz1*], *Arg1*, macrophage mannose receptor 1 [*Mrc1*], and *Ym1* [chitinase-like protein 3]) in the transfection group than in the induction group (**Figure 3A**). F4/80 and CD206 were detected as M2a macrophage markers. Our flow cytometry results showed that the proportion of M2a macrophages decreased compared with that in the induction group (**Figure 3B**). Previous data demonstrated that emu-miR-10a-5p was encapsulated in *E. multilocularis* EVs (22), which was confirmed in this study (**Supplementary Figures S1A, B**). In addition, *E. multilocularis* EVs can increase the expression of emu-miR-10a-5p in macrophages (**Supplementary Figure S1C**), indicating that *E. multilocularis* EVs can deliver emu-miR-10a-5p to macrophages.

MH-S cells were transfected with emu-miR-10a-5p mimics or NC mimics and cultured in the presence of IL-4 and IL-13 to promote M2a polarization. Our RT-qPCR results showed that miR-10a-5p expression was higher in the induction group than in the control group and that the expression of M2a genes was higher in the induction group (**Figures 3C, D**). Our flow cytometry results showed that the proportion of M2a macrophages was higher in the induction group (**Figure 3E**).

Overall, these results confirmed that *E. multilocularis* EVs and encapsulated emu-miR-10a-5p inhibited macrophage polarization towards the M2a phenotype.

### emu-miR-10a-5p might regulate M2a polarization in macrophages by targeting LIF and its pathways

**Figure 4:**
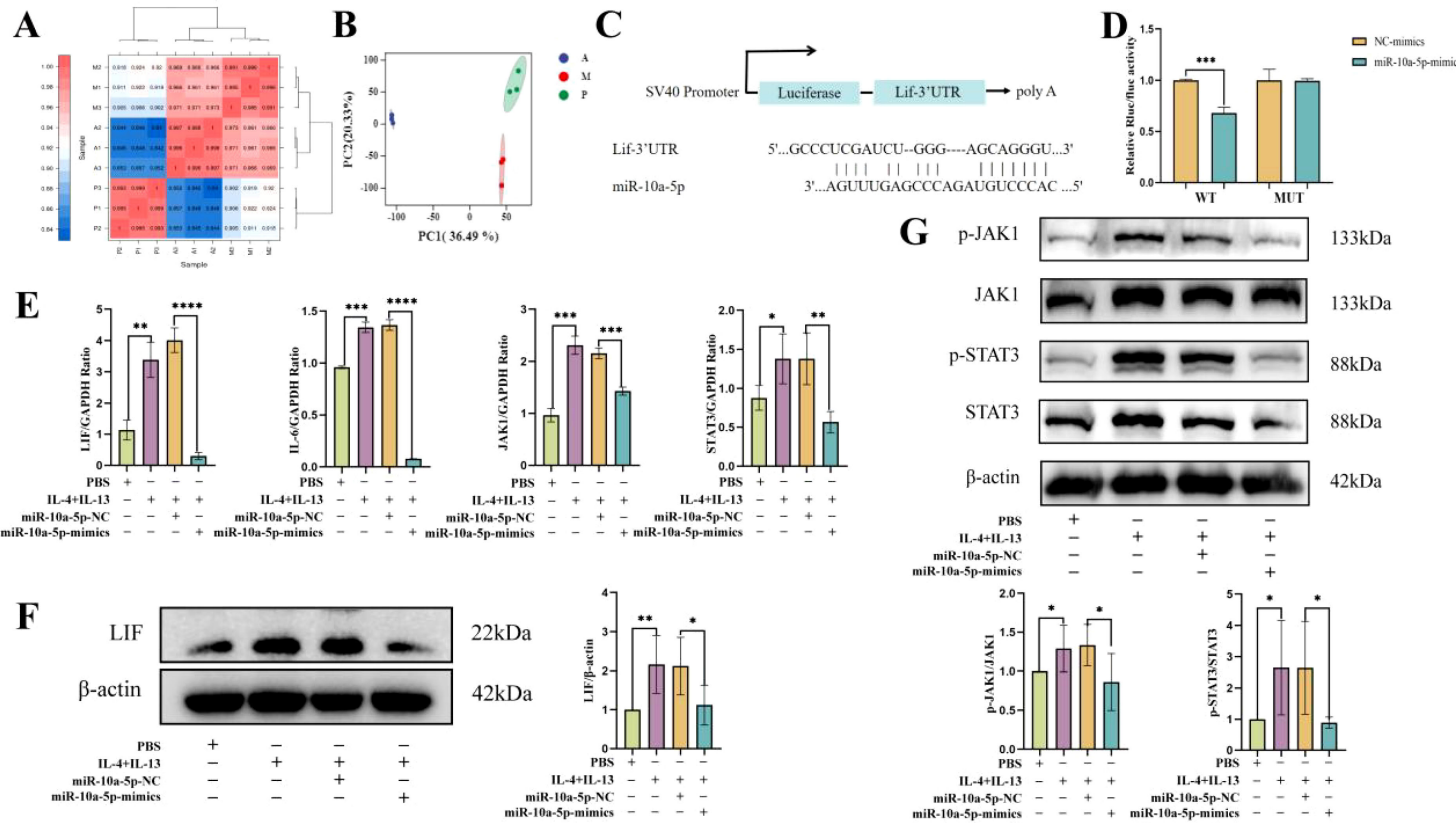
emu-miR-10a-5p regulated the JAK1–STAT3 signaling pathway by targeting LIF. Transcriptome sequencing was used to analyze gene expression. P represents the blank group, M represents the M2a-polarized group, and A represents the M2a-polarized group transfected with emu-miR-10a-5p. **(A)** Repeat correlation assessment. **(B)** Principal component analysis. **(C)** Binding site of emu-miR-10a-5p on LIF. **(D)** Luciferase activity was observed after emu-miR-10a-5p–LIF binding, as measured in dual-luciferase reporter assays. **(E)** mRNA-expression levels of *Lif*, *IL-6*, *Jak1*, and *Stat3* in macrophages transfected with emu-miR-10a-5p mimics, as determined using RT-qPCR. **(F)** LIF expression in macrophages transfected with emu-miR-10a-5p mimics was determined using western blotting. **(G)** JAK1 and STAT3 expression in macrophages transfected with emu-miR-10a-5p mimics, as determined using western blotting. **(E)** n=9, **(F)** and **(G)** n=5-6. *P < 0.05, **P < 0.01, ***P < 0.001, ****P < 0.0001.

To further investigate how emu-miR-10a-5p regulates macrophage M2a polarization, we screened emu-miR-10a-5p target genes via transcriptome sequencing. The results of repeat correlation assessment (**Figure 4A**) and principal component analysis (**Figure 4B**) indicated that the sequenced samples showed tight clustering, with high intragroup similarity and good repeatability. Comparison with the blank group revealed 248 upregulated genes in the M2a-polarized group. Comparison with the M2a-polarized group showed 224 downregulated genes after transfection with emu-miR-10a-5p (**Supplementary Figure S2A, B**). Comparison with the blank group indicated that *Mrc1* was upregulated in the M2a group and downregulated after transfection with emu-miR-10a-5p, consistent with our RT-qPCR results. Next, we conducted Kyoto Encyclopedia of Genes and Genomes and Gene Ontology analyses with the identified differentially expressed genes (**Supplementary Figures S2C–F**). We focused on highly enriched pathways, such as “immune response” and “cytokine-cytokine receptor interaction.” The *Lif* gene showed the most significant differences and was selected as a candidate target gene for emu-miR-10a-5p.

Our dual-luciferase assay results showed that co-transfecting emu-miR-10a-5p mimics significantly inhibited the luciferase activity of the LIF wild-type 3'-UTR constructs in 293T cells (**Figures 4C, D**). LIF can activate the JAK–STAT3 pathway by inducing the heterodimerization between the LIF receptor and the signal-transduction protein, gp130 (28); JAK1 is the kinase initially targeted by LIF (29). In addition, mounting evidence suggests that the JAK1–STAT3 signaling pathway participates in M2 macrophage polarization (30, 31). We examined the effect of emu-miR-10a-5p on the LIF and JAK1–STAT3 signaling pathways in MH-S cells. RT-qPCR and western blotting data showed that emu-miR-10a-5p significantly inhibited LIF expression and JAK1–STAT3 signaling in M2a macrophages (**Figures 4E–G**).

These findings indicate that emu-miR-10a-5p inhibited the JAK1–STAT3 signaling pathway by the targeted inhibition of LIF.

### LIF modulated M2a macrophage polarization via the JAK1–STAT3 signaling pathway

**Figure 5:**
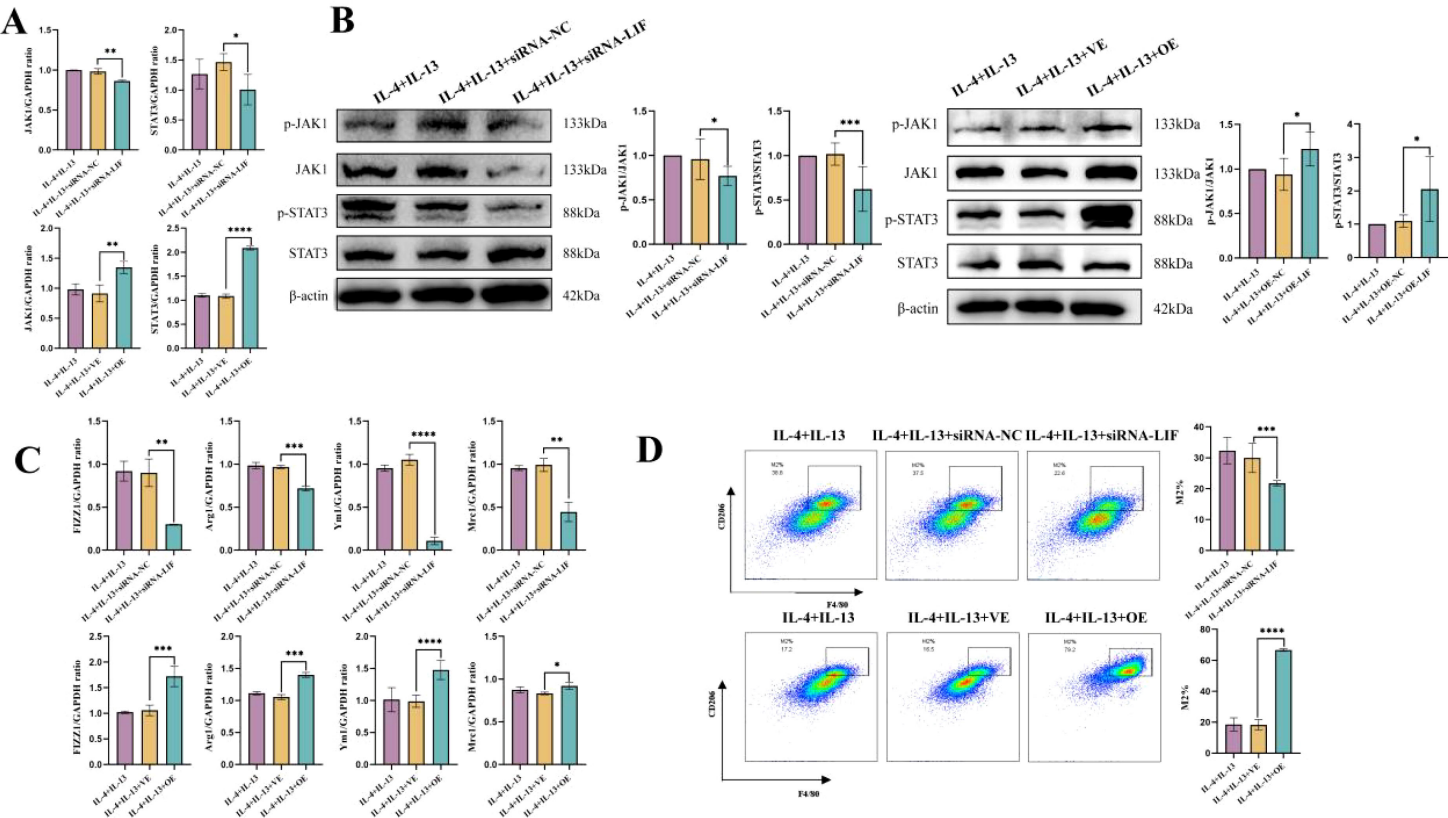
LIF modulated M2a macrophage polarization via the JAK1–STAT3 signaling pathway **(A)** *Jak1* and *Stat3* mRNA-expression levels in macrophages were determined via RT-qPCR after OE-LIF infection or siRNA-LIF transfection. **(B)** The protein levels of p-JAK1, JAK1, p-STAT3, and STAT3 in macrophages after OE-LIF infection or siRNA-LIF transfection were assessed via western blotting. **(C)** *Fizz1*, *Arg1*, *Mrc1*, and *Ym1* mRNA-expression levels in macrophages after OE-LIF infection or siRNA-LIF transfection were determined via RT-qPCR analysis. **(D)** Flow cytometry was used to detect the indicated proteins in macrophages after OE-LIF infection or siRNA-LIF transfection. Except **(B)** n=5-6, the remaining experiments n=9. *P < 0.05, **P < 0.01, ***P < 0.001, ****P < 0.0001.

To further clarify whether emu-miR-10a-5p regulates macrophage M2a polarization via LIF–JAK1–STAT3 signaling, we first infected cells with lentiviruses overexpressing LIF (OE-LIF) or transfected them with LIF siRNA. We successfully silenced or overexpressed LIF, and the LIF siRNA-346 fragment was more effective than other siRNAs tested (**Supplementary Figure S3**). Therefore LIF siRNA-346 was used in subsequent experiments, referred to simply as siRNA LIF. Comparison with the siRNA-NC group showed that the siRNA-LIF group had decreased JAK1 and STAT3 expression at 48 h post-transfection. Opposite results were observed in the transfected OE-LIF group (**Figures 5A, B**). The expression of M2a genes and the proportion of M2a macrophages were lower after siRNA-LIF transfection than after siRNA-NC transfection, whereas the opposite results were observed with the transfected OE-LIF group (**Figures 5C, D**). In conclusion, LIF promoted JAK1–STAT3 signaling and modulated M2a polarization in macrophages.

### Combined effects of emu-miR-10a-5p and LIF on M2a macrophage polarization and JAK1–STAT3 signaling

**Figure 6:**
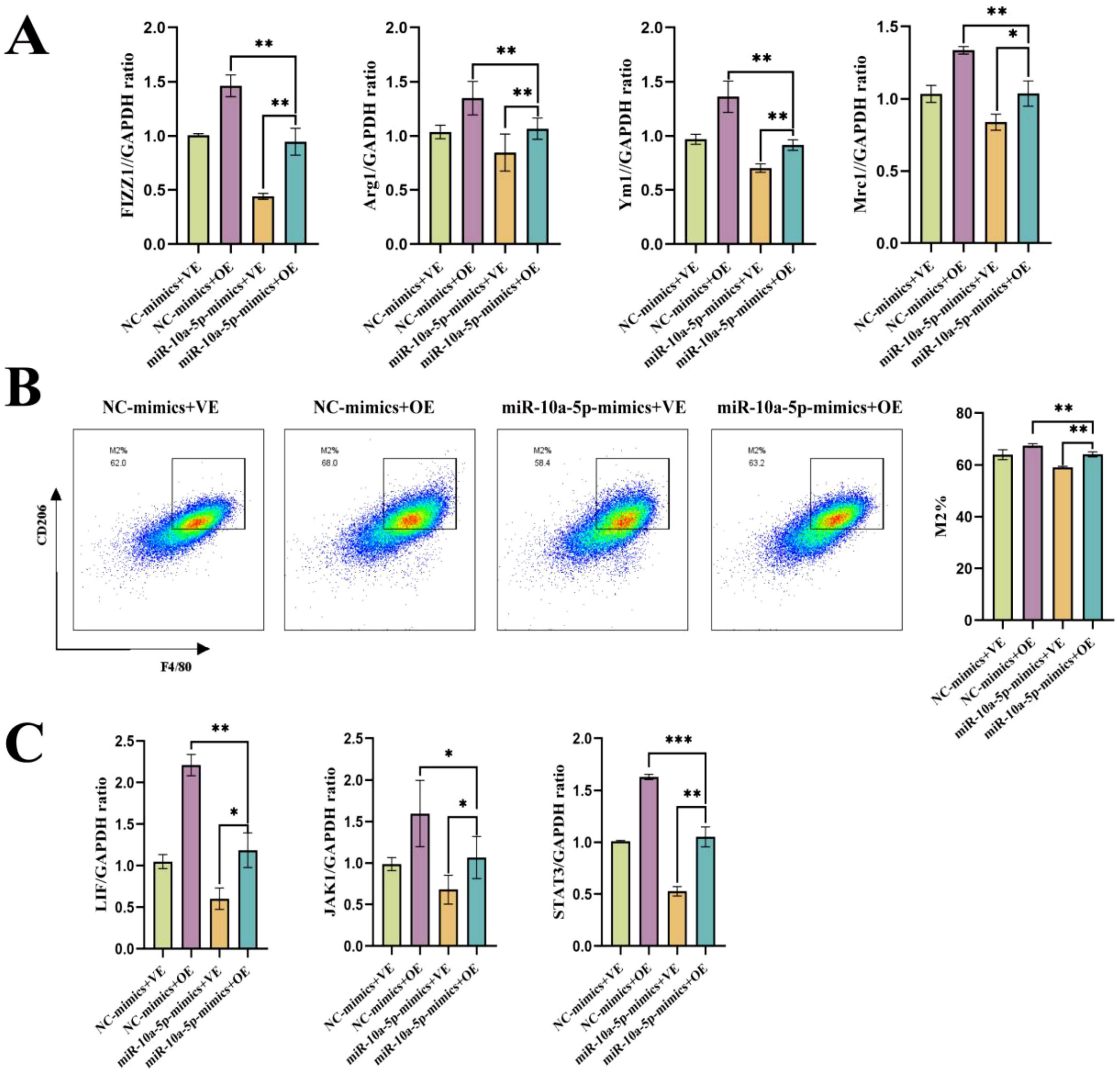
emu-miR-10a-5p and LIF together can rescue their individual effects on M2a macrophage polarization. **(A)** mRNA-expression levels of *Fizz1*, *Arg1*, *Mrc1*, and *Ym1* in macrophages transfected with emu-miR-10a-5p mimics and infected with an OE-LIF lentivirus were determined via RT-qPCR. **(B)** Flow cytometry was used to measure the expression of the indicated proteins in macrophages transfected with emu-miR-10a-5p mimics and infected with an OE-LIF lentivirus. **(C)** mRNA-expression levels of *Lif*, *Jak1*, and *Stat3* in macrophages transfected with emu-miR-10a-5p mimics and infected with an OE-LIF lentivirus were measured via RT-qPCR. All experiments n=9. *P < 0.05, **P < 0.01, ***P < 0.001.

To further verify the effects of emu-miR-10a-5p and LIF on macrophage M2a polarization, we co-transfected macrophages with emu-miR-10a-5p mimics and OE-LIF (with NC mimics + OE-NC as controls) and assessed M2a levels. RT-qPCR and flow cytometric analysis demonstrated that the emu-miR-10a-5p mimics+OE-LIF group reversed their individual effects, reaching levels similar to the NC-mimics+OE-NC group (**Figures 6A, B**). In agreement, RT-qPCR analysis demonstrated that the combined treatment alleviated alterations in the JAK1–STAT3 pathway (**Figure 6C**). These results suggest that emu-miR-10a-5p, in combination with LIF, affect M2a macrophage polarization through the LIF–JAK1–STAT3 axis.

### emu-miR-10a-5p relieved airway inflammation in mice with allergic asthma

**Figure 7:**
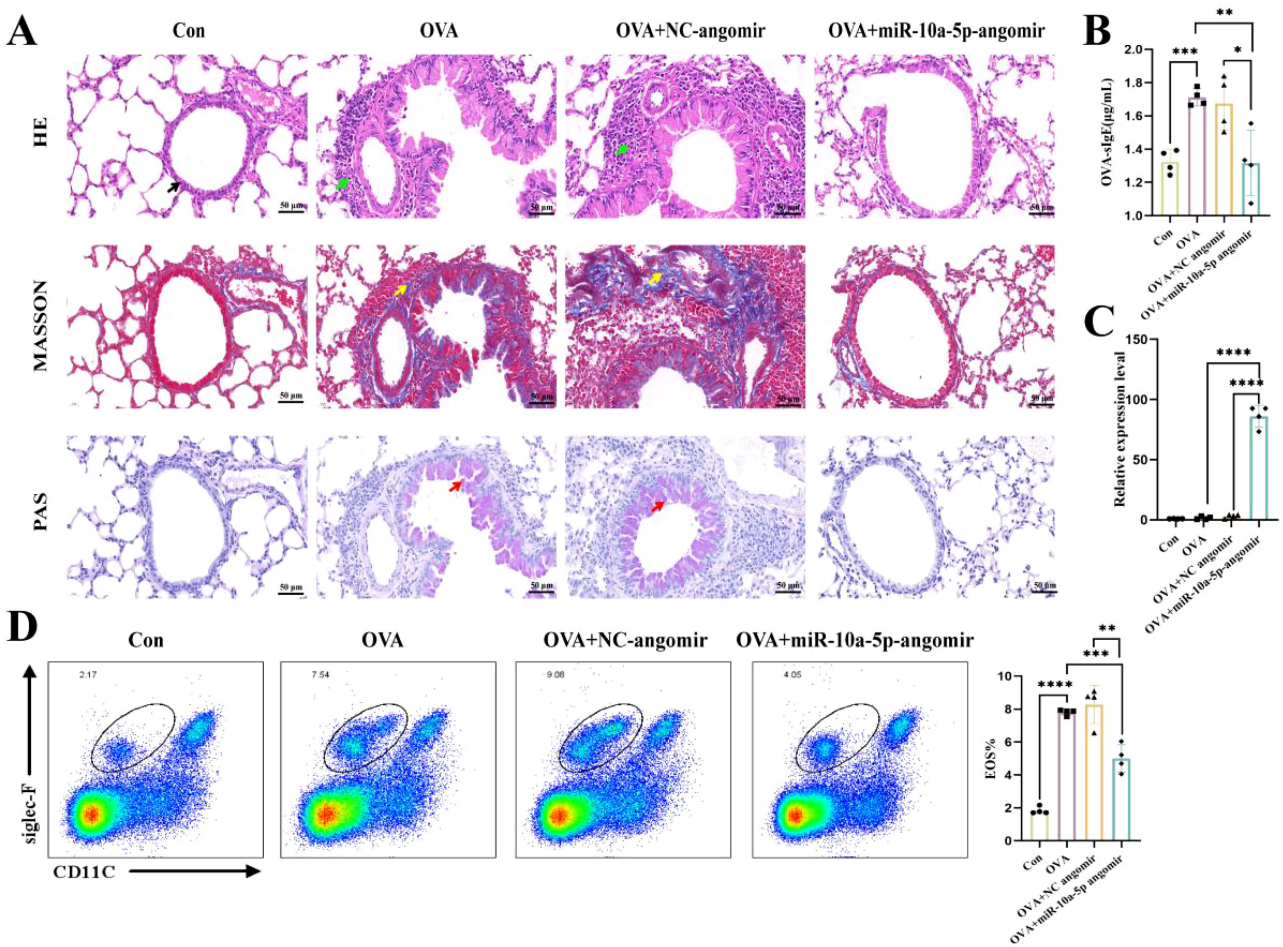
emu-miR-10a-5p relieved airway inflammation in mice with allergic asthma. **(A)** Morphological observations of H&E, Masson’s, and PAS staining in mouse lung tissues. Magnification: 40×, scale bar: 50 μm. The black arrows point to airway epithelial cells, the green arrows point to inflammatory cells, the yellow arrows point to blue-stained collagen fibers, and the red arrows point to proliferating goblet cells. **(B)** OVA-specific IgE expression in mouse serum was determined via ELISA analysis. **(C)** emu-miR-10a-5p expression in mouse lungs was determined via RT-qPCR analysis. **(D)** Flow cytometry was used to detect eosinophils in mouse lung tissues. All experiments n=4-6. *P < 0.05, **P < 0.01, ***P < 0.001, ****P < 0.0001.

Next, we established a mouse model of allergic asthma to investigate the role of emu-miR-10a-5p. H&E staining showed that mice in the OVA and OVA+NC agomir groups had thicker airway epithelia and more inflammatory cell infiltration around the bronchus than the control group. Masson’s staining showed significantly more hyperplasia of peribronchial collagen fibers in OVA and OVA+NC agomir mice than in the control group. PAS staining showed substantially more mucus secretion in the OVA and OVA+NC agomir groups than in the control group. These pathological changes were alleviated to a certain extent after emu-miR-10a-5p intervention (**Figure 7A**). Serum-specific IgE and pulmonary eosinophil levels increased in mice with allergic asthma and decreased after emu-miR-10a-5p intervention (**Figures 7B, D**). We also detected emu-miR-10a-5p in mouse lung tissues, finding that its expression only increased in the OVA+emu-miR-10a-5p-agomir group, indicating the successful tail vein intervention (**Figure 7C**). These results suggested that emu-miR-10a-5p relieved airway inflammation in mice with allergic asthma.

### emu-miR-10a-5p inhibited LIF–JAK1–STAT3 signaling and M2a macrophage polarization in lung macrophages from mice with allergic asthma

**Figure 8:**
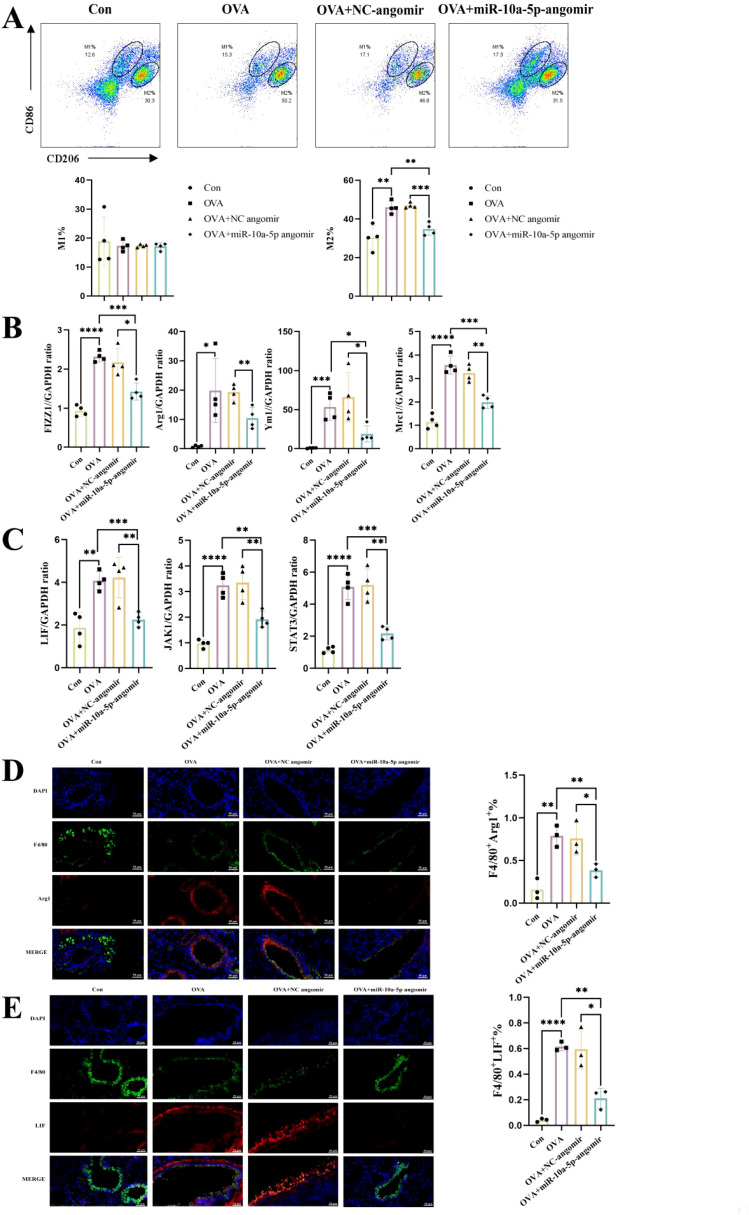
emu-miR-10a-5p inhibited LIF–JAK1–STAT3 signaling in lung macrophages from mice with allergic asthma and inhibited M2a polarization. **(A)** Flow cytometry was used to detect M1 and M2 macrophages in mouse lung tissues. **(B)** The mRNA-expression levels of *Fizz1*, *Arg1*, *Mrc1*, and *Ym1* in mouse lungs were determined via RT-qPCR. **(C)** The mRNA-expression levels of *Lif*, *Jak1*, and *Stat3* in mouse lungs were determined via RT-qPCR. **(D)** M2a macrophages in mouse lungs were detected via IF analysis (magnification: 40×, scale bar: 50 μm). **(E)** LIF expression in mouse lung macrophages was detected via IF analysis (magnification: 40×, scale bar: 50 μm). All experiments n=4-6.

To elucidate whether emu-miR-10a-5p might alleviate allergic asthma by inhibiting macrophage M2a polarization, we examined changes in the macrophage phenotypes in the lungs of various groups of mice. We observed no significant difference in the proportion of M1-type macrophages in the lung tissues among all treatment groups; however, M2a macrophages and associated genes were upregulated in allergic asthmatic mice but downregulated after emu-miR-10a-5p intervention (**Figures 8A, B, D**). Our RT-qPCR results showed that *Lif*, *Jak1*, and *Stat3* transcription increased in lung tissues from mice with allergic asthma and decreased after emu-miR-10a-5p intervention (**Figure 8C**). Immunofluorescence analysis showed that LIF expression in lung macrophages from allergic asthmatic mice increased and decreased after emu-miR-10a-5p intervention (**Figure 8E**). These results are consistent with those of our *in vitro* experiments, suggesting that emu-miR-10a-5p inhibited M2a macrophage polarization in lungs via the LIF–JAK1–STAT3 axis *in vivo*.

In addition, we sought to explore whether the intervention of emu-miR-10a-5p can correct the Th1/2 imbalance in the lung and spleen of mice with allergic asthma. (**Supplementary Figures S4A–D**). To further explore how emu-miR-10a-5p affects CD4^+^ T cell differentiation, we transfected macrophages with emu-miR-10a-5p-mimics and co-cultured them with mouse spleen CD4^+^ T cells. Flow cytometry showed that co-culturing naïve CD4^+^ T cells with M2a macrophages tended to promote Th2 cell differentiation and inhibit Th1 cell differentiation, whereas co-culturing naïve CD4^+^ T cells with macrophages transfected with emu-miR-10a-5p showed the opposite effects (**Supplementary Figures S4E, F**).

## Discussion

Building on the hygiene hypothesis, interest in studying parasite regulation of the immune system has been increasing. Some parasites and their products can protect the host from immune diseases, such as allergies, immune deficiency, and metabolic syndrome, either directly or by suppressing the inflammatory response (32). Several derivatives of parasite can relieve allergic OVA-induced asthma (32–35). Parasite miRNAs and antigens carried by parasite-derived exosomes play crucial roles in exchanging information and host–parasite interactions. *E. granulosus* PSC-derived exosome-like vesicles can be internalized by bone marrow-derived DCs to deliver egr-miR-277a-3p, which modulates the host immune response (21). However, few studies have been conducted on parasite-derived exosomes and the parasitic miRNAs they carry in allergic asthma.

emu-miR-10a-5p was highly expressed in EVs secreted by *E. multilocularis* (22), and interestingly, its sequence was identical to that of *E. granulosus* egr-miR-10a-5p. *E. granulosus* EVs showed high expression of egr-miR-10a-5p (36). In addition, sera from *E. granulosus*-infected mice contained emu-miR-10a-5p (23), suggesting that emu-miR-10a-5p likely helps regulate host cell immune responses. In this study, we demonstrated that *E. multilocularis* EVs and encapsulated emu-miR-10a-5p inhibited M2a macrophage polarization. In addition, emu-miR-10a-5p alleviated OVA-induced airway inflammation (including reducing eosinophilic infiltration into the airway and decreasing serum IgE levels) by inhibiting macrophage polarization toward the M2a phenotype. We searched for downstream target mRNA molecules via transcriptome sequencing to further investigate how emu-miR-10a-5p can inhibit M2a macrophage polarization in allergic asthma. We screened the candidate molecule LIF and verified its binding with emu-miR-10a-5p.

LIF is a member of the IL-6 cytokine family that plays important roles in homeostasis and disease, primarily through the JAK–STAT, mitogen-activated protein kinase–extracellular signal-regulated kinase and phosphoinositide 3-kinase (PI3K) pathways. Mounting evidence suggests that LIF is associated with the development of asthma (37–39), LIF might function as a proinflammatory cytokine in the airways by augmenting eosinophil recruitment and activation (40). Evidence exists that LIF promotes M2 macrophage polarization through STAT3 (41, 42). In this study, we demonstrated for the first time that LIF regulated M2a polarization of mouse alveolar macrophages (MH-S cells) via JAK1–STAT3 signaling. Moreover, we showed that emu-miR-10a-5p targeted LIF to inhibit macrophage M2a polarization through the same pathway, ultimately alleviating airway inflammation in allergic asthmatic mice. These results help us understand the roles of LIF in macrophage polarization and asthma pathogenesis.

Abnormal regulation of hsa-miR-10a-5p in bronchial epithelial cells may be an important mechanism underlying asthma (24). hsa-miR-10 can prevent airway smooth muscle cell proliferation by inhibiting PI3K signaling, making PI3K a potential target for treating lung diseases such as asthma (43). mmu-miR-10a-5p was upregulated in the exosomes of bronchoalveolar lavage fluid from asthmatic mice, promoted lung epithelial cell proliferation in mice, and downregulated Nfat5 and Map2k6 (25). Elevating mmu-miR-10a-5p levels inhibited proinflammatory gene expression in RAW264.7 macrophages and promoted the differentiation of C3H10T1/2 cells into brown adipocytes (44). And mmu-miR-10a-5p has strong potential for clinical application of cancer treatment (45). The seed sequences of emu-miR-10a-5p, hsa-miR-10a-5p, and mmu-miR-10a-5p were identical (5'-ACCCUGUA-3'), suggesting that emu-miR-10a-5p can potentially alter host mRNA expression. mmu-miR-10a-5p can act on IL-6R and thus reduce IL-6-induced cartilage cell ferroptosis (46). We also observed in our study that emu-miR-10a-5p can reduce IL-6 expression.

In this study, macrophages transfected with emu-miR-10a-5p tended to induce naïve CD4^+^ T cell differentiation to Th1 cells, and emu-miR-10a-5p tended to reverse the Th1/Th2 balance in mice with allergic asthma, consistent with our *in vitro* results. However, these differences were not statistically significant. These observations suggest that the alleviating effect of emu-miR-10a-5p on allergic asthma may occur primarily through the modulation of macrophage polarization or that other immune cells (such as DCs) may play a role in the Th1/Th2 balance; however, the specific mechanisms should be studied further.

## Conclusion

**Figure 9:**
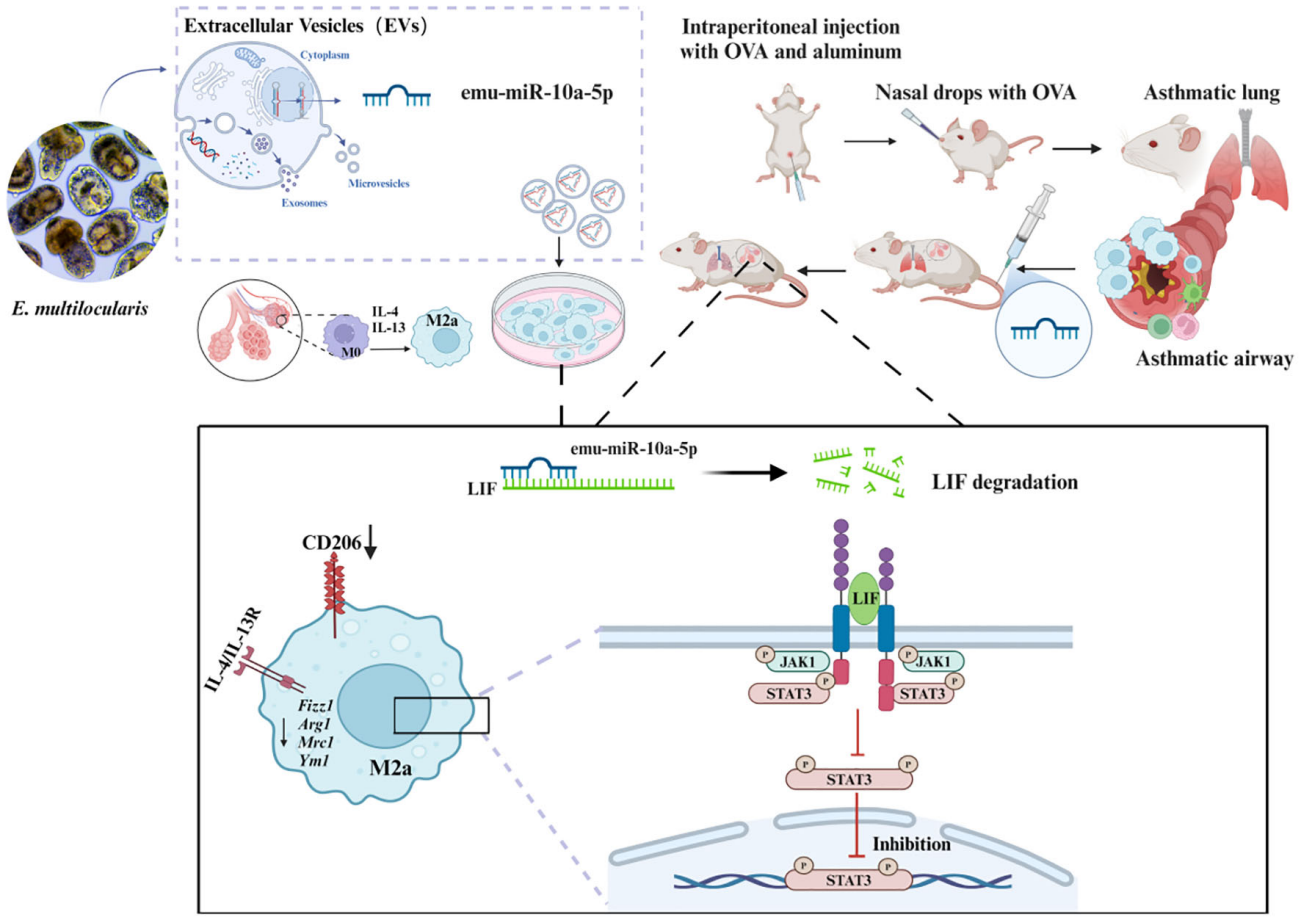
Schematic representation of the mechanism by which *E. multilocularis*-derived EVs could alleviate macrophage M2a polarization and that encapsulated emu-miR-10a-5p can target LIF and inhibit macrophage M2a polarization via JAK1-STAT3 signaling, subsequently alleviating airway inflammation in OVA-induced allergic asthma mice.

In conclusion, our findings demonstrated that *E. multilocularis*-derived EVs could alleviate macrophage M2a polarization and that encapsulated emu-miR-10a-5p can target LIF and inhibit macrophage M2a polarization via JAK1–STAT3 signaling, subsequently alleviating airway inflammation in OVA-induced allergic asthma mice (**Figure 9**).

## Data Availability

The datasets presented in this study can be found in online repositories. The names of the repository/repositories and accession number(s) can be found in the article/**Supplementary Material**.
