# Supplementary material for: Emu-miR-10a-5p in Echinococcus multilocularis-derived-extracellular vesicles alleviates airway inflammation in mice with allergic asthma by inhibiting macrophage M2a polarization through LIF-mediated JAK1–STAT3 signaling
Source: Front Immunol. 2025 May 27;16:1577349. doi: 10.3389/fimmu.2025.1577349 (PMC12149114; doi:10.3389/fimmu.2025.1577349)
Supplement: Supplementary file 1 [file DataSheet1.docx]

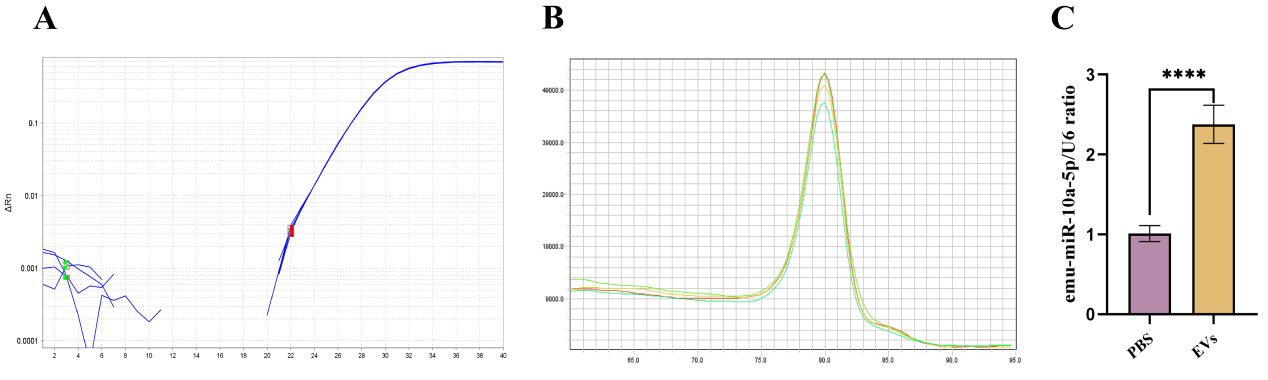


**Supplementary Fig. 1** Emu-miR-10a-5p was encapsulated in *E. multilocularis* EVs. (**A**) Amplification curve. (**B**) Dissolution curve. (C) *E.multilocularis*-derived- EVs impact on the emu-miR-10a-5p expression in macrophage was detected by RT-qPCR. All experiments n=3.


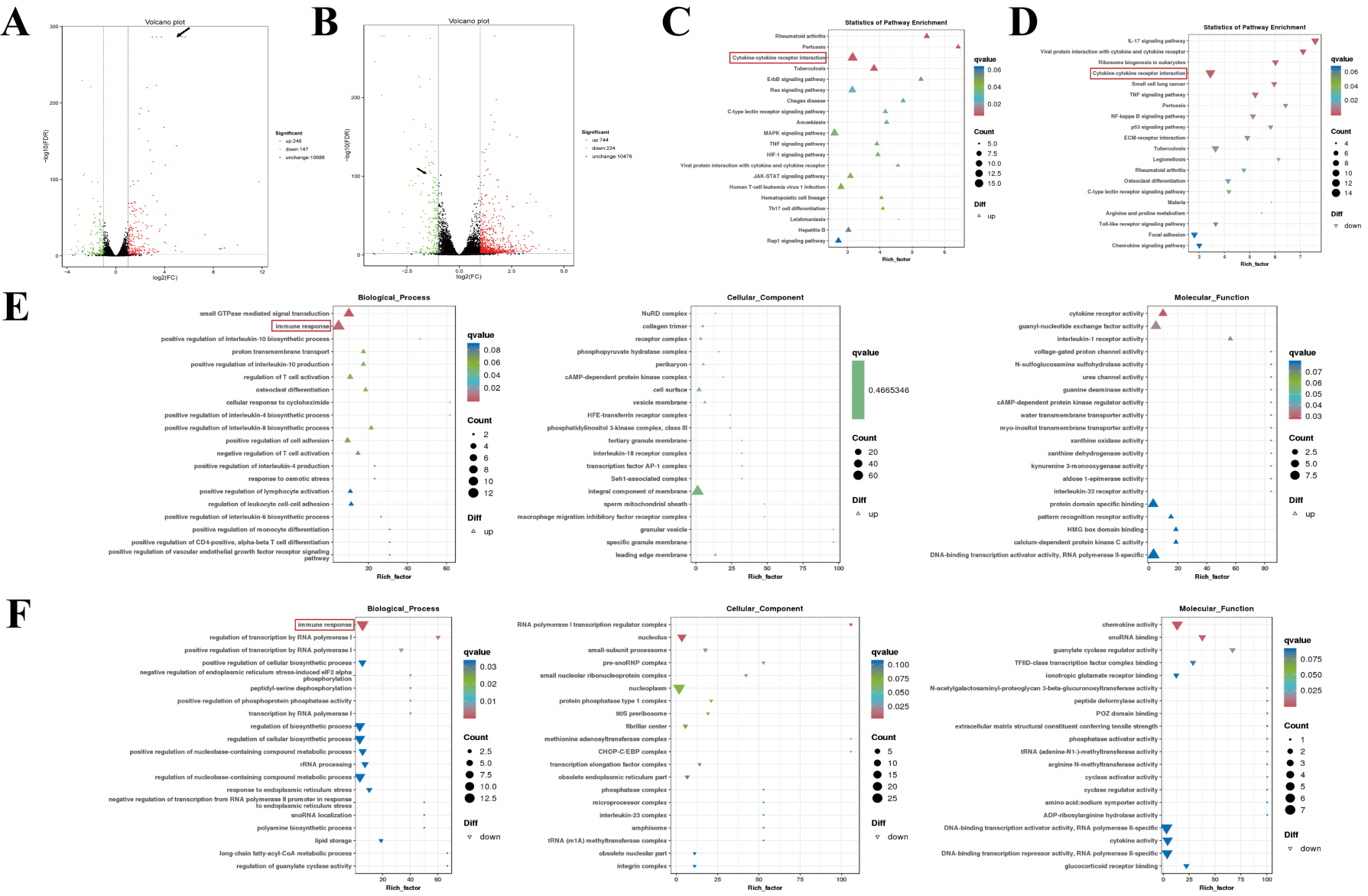


**Supplementary Fig. 2** Transcriptome sequencing. (**A**) Volcanic map of P vs M differential gene. (**B**) Volcanic map of M vs A differential gene. (**C**) KEGG annotated bubble map of P vs M up-regulated differential gene. (**D**) KEGG annotated bubble map of M vs A down-regulated differential gene. (**E**) GO annotated bubble map of PvsM up-regulated differential genes. (**F**) GO annotated bubble map of M vs A down-regulated differential genes. All experiments n=3.


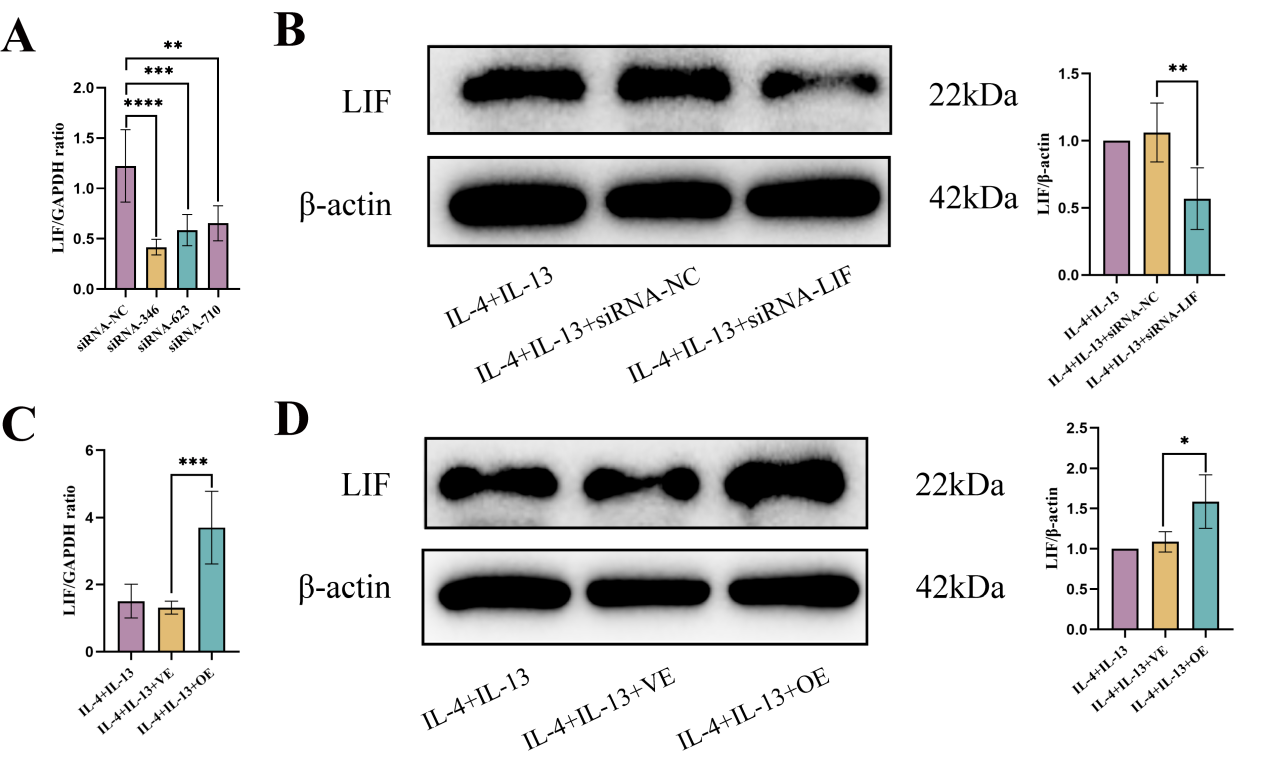


**Supplementary Fig. 3** Validation of the level of LIF silence or overexpression. (**A**) The silencing effect at the gene level was detected by RT-qPCR. (**B**) The silencing effect at the protein level was detected by Western blot. (**C**) The overexpression effect at the gene level was detected by RT-qPCR. (**D**) The overexpression effect at the protein level was detected by Western blot. All experiments n=5-6.


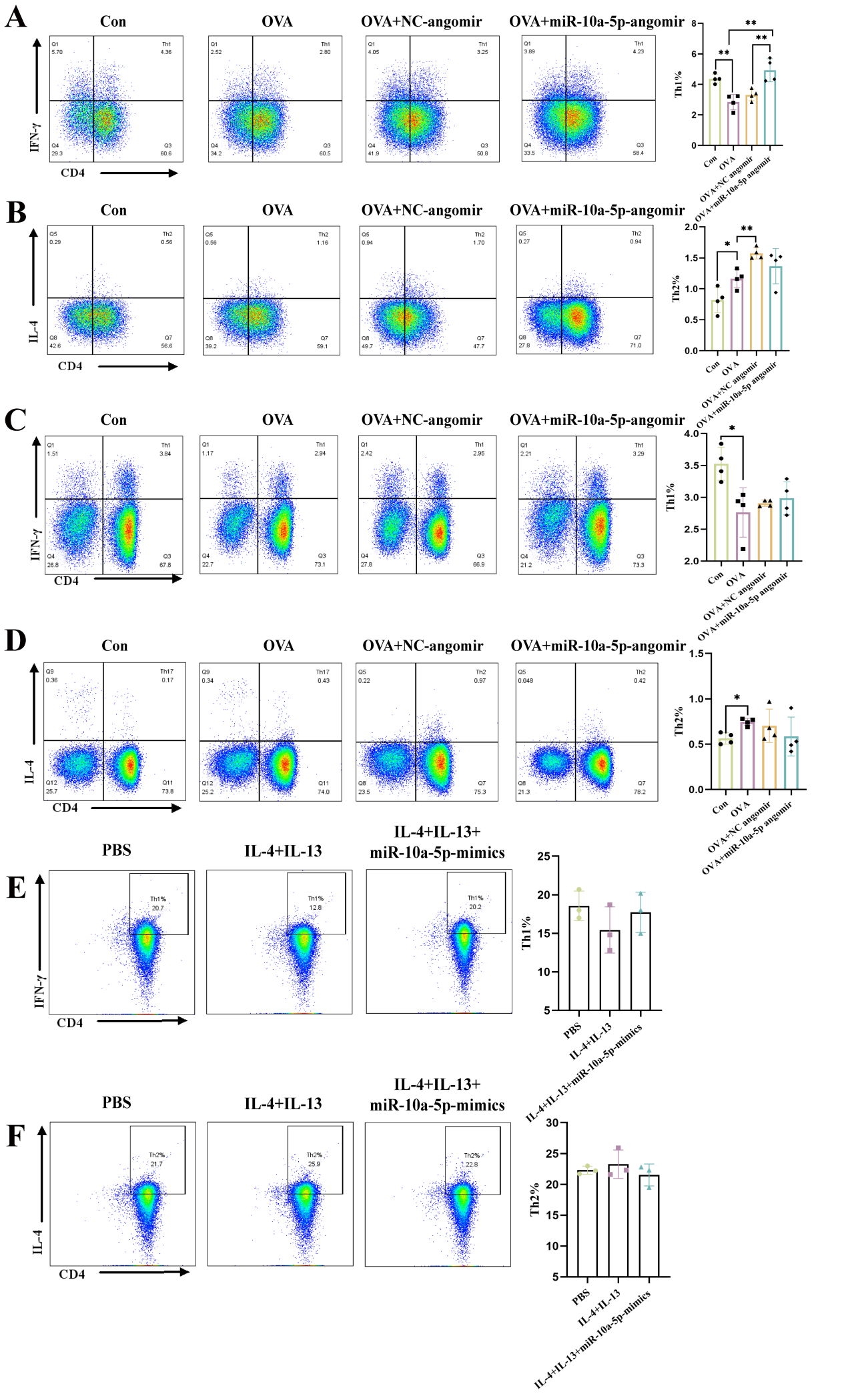


**Supplementary Fig. 4** Changes in Th1/Th2 balance in various groups of mice. (**A**) Flow cytometry was used to detect the expression of Th1 cells in mouse lung tissue. (**B**) Flow cytometry was used to detect the expression of Th2 cells in mouse lung tissue. (**C**) Flow cytometry was used to detect the expression of Th1 cells in mouse spleen tissue. (**D**) Flow cytometry was used to detect the expression of Th2 cells in mouse spleen tissue. (**E**) Flow cytometry was used to detect Th1 differentiation of CD4^+^T cells after co-culture with macrophages transfected with emu-miR-10a-5p. (**F**) Flow cytometry was used to detect Th2 differentiation of CD4^+^T cells after co-culture with macrophages transfected with emu-miR-10a-5p. All experiments n=3-6.

**Supplementary Table 1 Sequences of primers involved in RT-qPCR**

| Gene | Forward (5’-3’) | Rorward (5’-3’) | |
| --- | --- | --- | --- |
| Emu-miR-10a-5p | CCGCACCCTGTAGACCCGAGTTTG | |  |
| *U6* | GCTTCGGCAGCACATATACTAA | | CGAATTTGCGTGTCATCCTT |
| *GAPDH* | GGTTGTCTCCTGCGACTTCA | | TGGTCCAGGGTTTCTTACTCC |
| *Arg1* | AGCTCTAATCTGCATGG | | ATGTACACGATGTCTTTGGCAGATA |
| *Ym1* | CCACAGGAGCAGGAATCATTGAC | | TTCTCCACCCTGTAGATCCGAATTTG |
| *Mrc1* | AGGGAAGAGAAGAAGATCCAG | | TGGGAGAAGATGAAGTCAAAC |
| *FIZZ1* | TGCCAACTGTCCTAAGAATGA | | GCACATGAGTCAGATTTCCAA |
| *LIF* | GCAACGGGACAGAGAAGACCAAG | | GCACAGACGGCAAAGCACATTG |
| *IL-6* | CTTCTTGGGACTGATGCTGGTGAC | | AGGTCTGTTGGGAGTGGTATCCTC |
| *JAK1* | ACTACCGGATGAGGTTCTACC | | GGGTCTCGAATAGGAGCCAG |
| *STAT3* | CAGCAGCTTGACACACGGTA | | AAACACCAAAGTGGCATGTGA |
